# The influence of exercise interventions on cognitive functions in patients with amnestic mild cognitive impairment: A systematic review and meta-analysis

**DOI:** 10.3389/fpubh.2022.1046841

**Published:** 2022-11-15

**Authors:** Rong Wang, Hanyue Zhang, Hongjuan Li, Hong Ren, Tingting Sun, Liya Xu, Yang Liu, Xiao Hou

**Affiliations:** ^1^Department of Physical Fitness and Health, School of Sport Science, Beijing Sport University, Beijing, China; ^2^Key Laboratory of Sports and Physical Health Ministry of Education, Beijing Sport University, Beijing, China; ^3^School of Physical Education, Northeast Normal University, Changchun, China; ^4^Department of Physical Education, Shandong Jianzhu University, Jinan, China

**Keywords:** exercise, cognitive function, mild cognitive impairment, amnestic, meta-analysis, physical activity

## Abstract

**Introduction:**

Patients with amnestic mild cognitive impairment (aMCI) are more likely to develop dementia compared to patients with non-aMCI (naMCI). Among the mixed samples of aMCI and naMCI, exercise interventions are effective for patients with MCI to improve cognitive functions. However, the influence of exercise interventions on patients with aMCI is still unclear.

**Objective:**

The objective of this systematic review and meta-analysis is to evaluate the influence of exercise interventions on cognitive functions in patients with aMCI.

**Methods:**

Four literature databases (PubMed, Web of Science, EBSCO, and Cochrane Library) and three Chinese databases (China National Knowledge Infrastructure, Wanfang, and China Science and Technology Journal Database) were searched from their inception to August 31, 2022. Based on the preliminary search of seven databases and their cited references, a total of 2,290 records were identified. Finally, 10 studies with a total of 28 data points involving 575 participants with aMCI were included in this meta-analysis. If the measurements of outcomes were different among studies, the effect size was synthesized using the standardized mean difference (SMD) with a 95% confidence interval (CI). If the measurements were the same, the weight mean difference (WMD) with a 95% CI was used to integrate the effect size.

**Data synthesis:**

The results showed that exercise interventions had no significant effects on improving several specific domains of cognitive functions including working memory (WMD = −0.05; 95% CI = −0.74 to 0.63; *p* = 0.88; *I*^2^ = 78%) and attention (SMD = 0.20; 95% CI = −0.31 to 0.72; *p* = 0.44; *I*^2^ = 60%). Additionally, exercise interventions had a significant effect on global cognitive function (SMD = 0.70; 95% CI = 0.50–0.90; *p* < 0.00001; *I*^2^ = 29%) and some specific cognitive domains including immediate recall (SMD = 0.55; 95% CI = 0.28–0.81; *p* < 0.0001; *I*^2^ = 0%), delayed recall (SMD = 0.66; 95% CI = 0.45–0.87; *p* < 0.00001; *I*^2^ = 37%), and executive function (SMD = 0.38; 95% CI = 0.16–0.60; *p*= 0.0006; *I*^2^ = 4%). Furthermore, subgroup analysis based on the intervention forms indicated that multi-component interventions (SMD = 0.44; 95% CI = 0.11–0.77; *p* = 0.009; *I*^2^ = 0%) appeared to be less effective than the single-component intervention (SMD = 0.85; 95% CI = 0.60–1.10; *p* < 0.00001; *I*^2^ = 10%) in terms of boosting global cognitive function.

**Conclusion:**

This meta-analysis suggests that the exercise can help patients with aMCI improve global cognitive function. And exercise interventions have positive influence on enhancing several specific cognitive domains such as immediate recall, delayed recall, and executive function.

**Systematic review registration:** http://www.crd.york.ac.uk/PROSPERO, identifier: CRD42022354235.

## Introduction

Recently, the population aging has become a global concern. World Health Organization (WHO) claims that the Western Pacific region has more than 245 million people over the age of 65 years and this number is projected to double by 2050 (1). The advanced age exacerbates the deterioration of the physical health and mental soundness of the elderly. Especially, the risk of developing dementia among older adults caused by aging is increasing rapidly. The estimated number of individuals with dementia has been predicted to reach 82 million worldwide by 2030 (2). The dementia severely impairs the quality of the life and the well-being of older adults and places a heavy burden on families and societies (3). Thus, there is an urgent need to find targeted treatments for slowing the progression of dementia.

In recent years, the increasing attention has been paid to populations with mild cognitive impairment (MCI) in order to prevent the occurrence of the dementia. MCI is a transitional condition that occurs between normal cognitive aging and dementia (4). According to the relevant data, the MCI prevalence in older adults between 60 and 84 years old is 6.7–25.2% (5). A meta-analysis (6) suggests that 12.2% of over 55-year-old Chinese people meet the diagnostic criteria for MCI and the elderly have a higher rate of MCI as their age increases. Patients who are diagnosed as MCI are worse in terms of the overall health and the balance than people without MCI (7). Also, it has been reported that patients with MCI are detected to have a higher chance of getting neuropsychiatric symptoms such as depression, anxiety, irritability, agitation, and apathy compared to healthy people (8, 9). Several studies have presented that MCI individuals who are accompanied by the symptoms of depression, apathy, and anxiety are more likely to develop Alzheimer's disease (AD) (10–12). In view of the above, it is vital to implement cost-effective and efficient treatments for MCI patients as early as possible.

Currently, there's no strong evidence supporting the prominent impact of drug treatments on improving cognitive functions in patients with MCI (13–16). Therefore, utilizing non-pharmacological interventions (e.g., exercise interventions) is a wise choice to increase cognitive levels for MCI individuals. Several original studies have demonstrated that exercise interventions are capable of enhancing cognitive functions (e.g., processing speed, working memory, and executive function) of MCI patients (17–19). For example, a 24-form simplified Tai Chi exercise intervention lasting 1 year contributed to a significant improvement in MCI older adults' delayed recall scores (20). Another study has illustrated that a 6-month resistance training is beneficial for the improvements of the global cognitive function in patients with MCI (21). Baker et al. (22) also have confirmed that the executive function of MCI patients can be ameliorated after a high-intensity aerobic training.

In addition, individuals with MCI can be further classified into amnestic MCI (aMCI) and non-amnestic MCI (naMCI) (23). Compared to naMCI people, patients with aMCI can suffer from more adverse influences in certain aspects. Several studies have proposed that individuals with aMCI have a greater risk of progressing to AD (24–26). Moreover, aMCI patients are more likely to obtain a higher hazard ratio of the death (27, 28). Consequently, we should pay more attention to the non-pharmacological interventions (e.g., exercise interventions) to raise the cognitive functions for aMCI patients.

Plenty of meta-analyses have focused on investigating the effect of different exercise interventions on cognitive functions in mixed samples of aMCI and naMCI (29–33). Specifically, Zhu et al. (31) in their meta-analysis highlighted that an aerobic dance intervention was useful to reinforce the global cognitive function, memory, and executive function of patients who were diagnosed with MCI. Another meta-analysis suggested that the twice-weekly resistance interventions could display a larger improvement in MCI individuals' global cognitive function but not for working memory (33). Although these meta-analyses have discussed the effect of exercise interventions on cognitive functions in mixed samples of MCI individuals, the researchers have not distinguished the subtypes of MCI including aMCI and naMCI. Hence, the separate influence of exercise interventions on the cognitive functions of aMCI individuals or naMCI individuals is still uncertain. Given that patients with aMCI get greater impairment in some aspects, it is important to integrate the relevant data using meta-analysis based on the original studies and comprehensively evaluate the effect of exercise interventions on aMCI patients' cognitive functions.

This systematic review and meta-analysis intends to explore the influence of exercise interventions on cognitive functions in patients with aMCI.

## Methods

### Search strategy and study selection

Based on the guidelines of the Preferred Reporting Items for Systematic Reviews and Meta-Analyses (PRISMA), we conducted this systematic review and meta-analysis by searching seven databases from their inception to August 31, 2022. PubMed (1978–2022), Web of Science (2010–2022), EBSCO (2005–2022), Cochrane Library (2012–2022), China National Knowledge Infrastructure (CNKI) (2006–2022), Wanfang (2000–2022), and China Science and Technology Journal Database (2016–2022) were searched respectively using several keywords. Two independent authors (RW and HZ) evaluated all titles, abstracts, and full-text articles to filter and identify relevant studies. In order to avoid missing related articles as much as possible, we also screened references and citations from the included studies. Any disagreement was settled by discussion or by consulting a third arbitrator (XH). Table 1 shows the specific search strategy in PubMed.

**Table 1:** Search strategy in PubMed.

**Step**	**Search strategy**
#1	“Cognit* impair*” OR “MCI” OR “aMCI” OR “memory” OR “amnestic” OR “cognition disorders” OR “cognitive dysfunction” OR “memory impairment” [Title/Abstract]
#2	“Cognition” OR “cognition disorders” OR “cognitive dysfunction” [Mesh]
#3	#1 OR #2
#4	“Exercise” OR “physical activit*” OR “aerobic training” OR “resistance training” OR “circuit-based exercise” OR “combined exercise” OR “Chinese traditional exercise” OR “Chinese exercise” OR “Baduanjin” OR “Tai chi” OR “Taiji*” OR “Qi gong” [Title/Abstract]
#5	“Exercise” OR “resistance training” OR “circuit-based exercise” [Mesh]
#6	#4 OR #5
#7	“Cognition” OR “executive function” OR “memory function” OR “working memory” OR “delayed recall” OR “immediate recall” OR “attention” OR “verbal fluency” OR “visuospatial ability” OR “processing speed” [Title/Abstract]
#8	“Randomized controlled trial” OR “controlled clinical trial” OR “clinical trial” [Publication Type]
#9	“Randomized controlled trials as topic” [Mesh]
#10	#8 OR #9
#11	#3 AND #6 AND #7 AND #10

We registered the protocol on the international prospective register of systematic reviews (http://www.crd.york.ac.uk/PROSPERO), registration number: CRD42022354235. There's no similar review protocol existing.

### Inclusion criteria

In accordance with the PICOS principle related to the terms of patient/population, intervention, comparison/control, outcome, and study design, trials that met all of the following criteria were included: (1) participants were over the age of 50 years old; (2) participants were screened or diagnosed with aMCI by neurologists or based on Petersen's criteria (23), or participants with MCI who had memory complaints; (3) participants in the experimental group received exercise interventions or exercise interventions combined with psychological cognitive interventions; (4) participants in the control group maintained their regular lifestyles, received educational classes, or conducted sham exercise interventions (e.g., stretching or toning); (5) the outcomes were the common indicators reflecting cognitive functions such as executive function, memory, or attention; (6) only trials designed as the randomized controlled trials (RCTs) were covered; and (7) the peer-reviewed articles.

### Exclusion criteria

Trials were excluded if they met one of the following exclusion criteria: (1) case reports, abstracts, or non-peer-reviewed articles (e.g., academic dissertations and conference posters); (2) trials recruited the mixed participants with different subtypes of MCI and the authors did not report the independent data points for aMCI patients; (3) participants were pregnant women, animals, or individuals who were diagnosed with other mental disorders like anxiety or schizophrenia; (4) participants in experimental and control groups received drug treatments; (5) missing data.

### Quality assessment

The Cochrane Collaboration tool was used by two authors to independently examine the seven domain biases: (1) random sequence generation (selection bias); (2) allocation concealment (selection bias); (3) blinding of participants and personnel (performance bias); (4) blinding of outcome assessment (detection bias); (5) incomplete outcome data (attrition bias); (6) selective reporting (reporting bias); and (7) other bias (34). Three levels of bias risk (i.e., high, low, and unclear) were applied to grade the included studies. Any disagreement was settled by discussion or by consulting a third arbitrator (XH).

### Data extraction

From each included study, two authors independently summarized the pertinent information as the following: author(s), country/region, participants' characteristics (e.g., age and gender), sample size, details of interventions (e.g., types, frequency, and duration), and reported outcomes.

### Meta-analysis

The meta-analysis was carried out using the Review Manager software (Review Manager 5.3; The Nordic Cochrane Center, The Cochrane Collaboration). In this meta-analysis, various cognitive functions were measured using different scales. For example, the global cognitive function was assessed by the Mini-Mental State Examination (MMSE), Montreal Cognitive Assessment (MoCA), and Alzheimer's Disease Assessment Scale-Cognitive subscale (ADAS-cog). The working memory was assessed by the Digit Span Test-Backward (DST-B). The immediate recall was evaluated by the Logical Memory I subtest of the Wechsler memory scale-revised (WMS-LM I) and Rivermead Behavioral Memory Test (RBMT). The delayed recall was evaluated by the Logical Memory II subtest of the Wechsler memory scale-revised (WMS-LM II) and RBMT. The executive function was appraised by the Symbol-Digit Substitution Test (SDST), Trail Making Test Parts B (TMT-B), Trail Making Test B minus A (TMT-B–A), and Stroop Color and Word Test (SCWT). The attention was appraised by the Digit Span Test-Forward (DST-F) and Test of Everyday Attention (TEA). If the reported outcomes were measured by different scales, the effect size would be synthesized using the standardized mean difference (SMD). If the outcomes were measured by the same scales, the effect size would be integrated using the weight mean difference (WMD). If outcomes were measured by reverse scored scales, the inverse of the original data would be used. Additionally, the Cochrane Handbook for Systematic Reviews stated that both post-intervention values (Mean _post−intervention_ ± SD _post−intervention_) of the outcome and changes from baseline (Mean _of changes_ ± SD _of changes_) could be employed to synthesize the effect size (35).

If studies presented standard error (SE) and the number of subjects (*N*), the formula “Standard Deviation (SD) = SE × √*N*” would be used to compute the SD. If studies provided a confidence interval (CI), the formula “SD = √*N* × (*l*_upper_-*l*_lower_)/c” would be used to determine the SD. The upper and lower limits of the CI were denoted by the *l*_upper_ and the *l*_lower_. And the constant c depended on the sample size and the CI (36). If studies did not provide the mean and SD of outcomes, we would contact the authors by email. The articles would be removed if we did not receive the reply.

The heterogeneity across the studies was evaluated using the *I*^2^ index. When *I*^2^ ≤ 25%, the low heterogeneity was estimated. When *I*^2^ ≤ 50% and >25%, the moderate heterogeneity was assessed. When *I*^2^ ≤ 75% and >50%, the high heterogeneity was identified. When *I*^2^ > 75%, the very high heterogeneity was evaluated (36). A random-effect model would be used to aggregate the outcomes for studies with high heterogeneity (*I*^2^ ≤ 75% and >50%) or very high heterogeneity (*I*^2^ > 75%). Otherwise, a fixed-effect model would be chosen. Possible publication bias was evaluated when *I*^2^ > 50% by assessing the asymmetry of funnel plots or by applying Egger's test (37). *p* < 0.05 was set as the significant level.

## Results

### Search results

**Figure 1:**
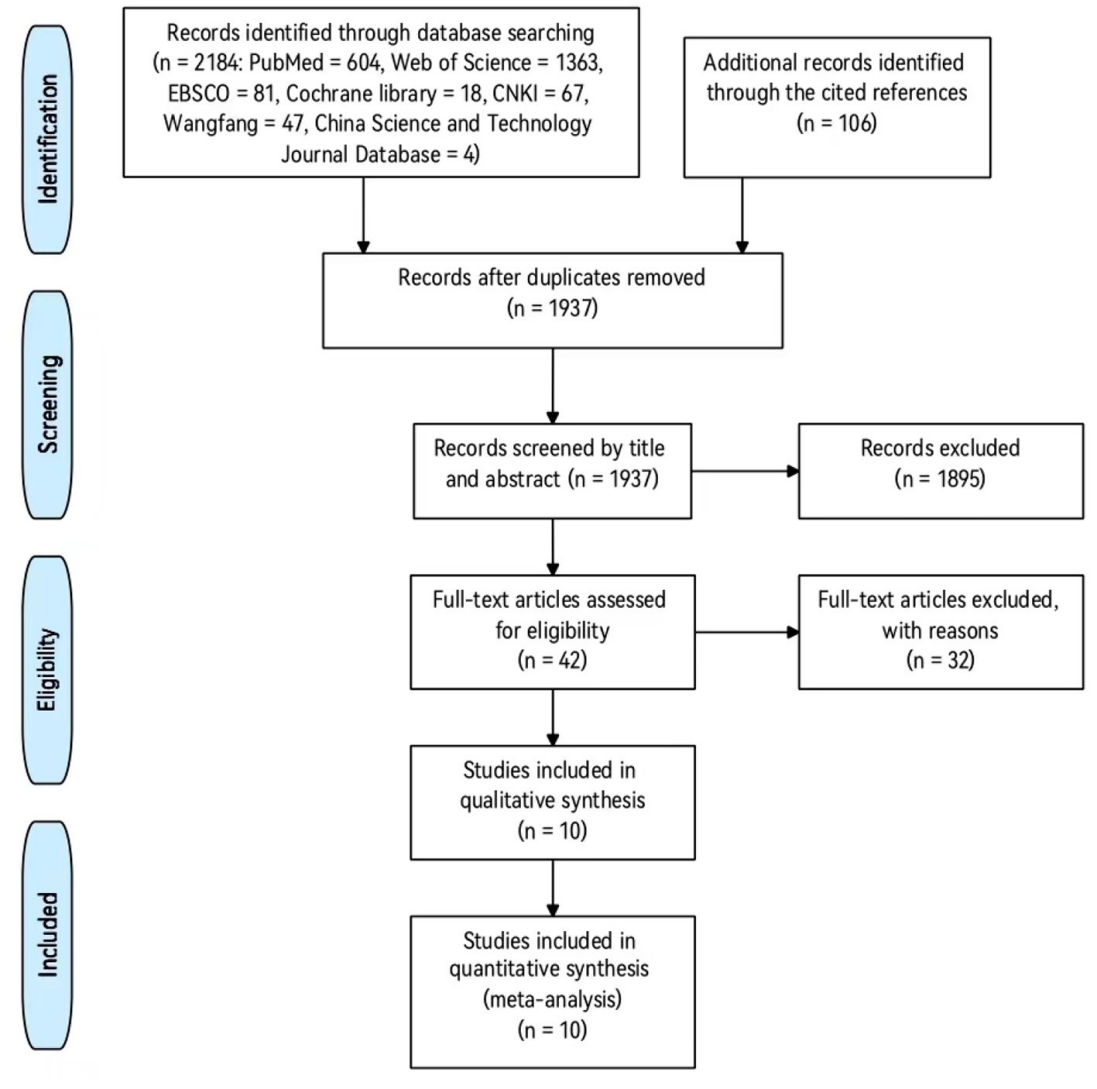
The flowchart of search procedure.

The search procedure is presented in Figure 1. A total of 2,290 records were found after our preliminary search of seven databases and their cited references. There were 1,937 records left after removing duplicates. There were still 42 eligible articles left after checking titles and abstracts. After examining the full-text papers, 10 articles qualified for inclusion. A total of 28 data points (covering 575 individuals) from the 10 articles were included in this meta-analysis.

### Characteristics of included studies

The characteristics of included studies are displayed in Table 2. The subjects in all studies were the aMCI older adults over the age of 50 years. These studies came from different countries around the world. One study (10%) was carried out in America (38). One study (10%) was conducted in China (39). One study (10%) was carried out in Greece (40). Two studies (20%) were performed in Japan (41, 42). Two studies (20%) were conducted in Korea (18, 43) and three studies (30%) were performed in Thailand (44–46).

**Table 2:** Characteristics of included studies.

**No**.	**Author (Ref)**	**Country**	**Participant age**	**The proportion of females (EG/CG)**	**Sample size**	**Intervention type**	**Intervention frequency**	**Intervention duration**	**Outcomes (measurements)**
1	Suzuki et al. (42)	Japan	Age: 65–92 years	48/44%	**EG:** 24 **CG:** 23	**EG:** multi-component intervention (AE, RE, postural balance retraining, dual-task training program: subjects performed concurrent cognitive tasks during exercise) **CG:** education classes	**EG:** 90 min/day; 2 days/week **CG:** two education classes over 6 months	6 months	Global cognitive function (MMSE); immediate recall (WMS-LM I); delayed recall (WMS-LM II)
2	Park et al. (43)	Korea	Age: 50–85 years	68/71%	**EG:** 25 **CG:** 24	**EG:** multi-component intervention (PA promotion and behavior modification, AE, dual-task training program: subjects performed concurrent cognitive tasks during exercise) **CG:** none	**EG:** 110 min/day; 2 days/week	24 weeks	Global cognitive function (MMSE); working memory (DST); executive function (SDST)
3	Hong et al. (18)	Korea	Age: >65 years	70/75%	**EG:** 10 **CG:** 12	**EG:** RE (10-min warm-up, 40-min RE at 65% of 1RM, 10-min cool-down) **CG:** maintain regular lifestyle	**EG:** 60 min/session; 2 sessions/week	12 weeks	Global cognitive function (MoCA); working memory (DST-B); attention (DST-F)
4	Lü et al. (39)	China	Age: ≥65 years	73/70%	**EG:** 22 **CG:** 23	**EG:** RE (5-min warm-up, 50-min dumbbell-spinning exercises, 5-min cool-down) **CG:** maintain regular lifestyle	**EG:** 60 min/session; 3 sessions/week	12 weeks	Global cognitive function (ADAS-cog); working memory (DST-B); attention (DST-F); executive function (TMT-B)
5	Suzuki et al. (41)	Japan	Age: 65–93 years	48/44%	**EG:** 25 **CG:** 25	**EG:** multi-component intervention (AE, RE, postural balance retraining, dual-task training program: subjects performed concurrent cognitive tasks during exercise) **CG:** education classes	**EG:** 90 min/day; 2 days/week **CG:** three education classes over 12 months	12 months	Global cognitive function (MMSE); immediate recall (WMS-LM I); delayed recall (WMS-LM II); executive function (SCWT)
6	Sungkarat et al. (45)	Thailand	Age: 65–92 years	94/79%	**EG:** 33 **CG:** 33	**EG:** TC (10-min warm-up, 30-min TC exercise, 10-min cool-down) **CG:** maintain regular lifestyle	**EG:** 50 min/session; 3 sessions/week	15 weeks	Delayed recall (WMS-LM II); executive function (TMT-B–A)
7	Lazarou et al. (40)	Greece	Mean age: 66.8 years	80/76%	**EG:** 66 **CG:** 63	**EG:** international ballroom dancing [5-min warm-up, 45 min of new material (figures/dances), 10-min cool-down] **CG:** maintain regular lifestyle	**EG:** 60 min/session; 2 sessions/week	40 weeks	Global cognitive function (MMSE); immediate recall (RBMT1); delayed recall (RBMT2); attention (TEA)
8	Sungkarat et al. (46)	Thailand	Mean age: 67.9 years	94/79%	**EG:** 33 **CG:** 33	**EG:** TC (10-min warm-up, 30-min TC exercise, 10-min cool-down) **CG:** education classes	**EG:** 50 min/session; 3 sessions/week **CG:** a 1-h presentation	6 months	Delayed recall (WMS-LM II); executive function (TMT-B–A)
9	Khanthong et al. (44)	Thailand	Age: 50–80 years	89/69%	**EG:** 35 **CG:** 36	**EG:** traditional Thai exercise (Ruesi Dadton: 15 postures with 10 repetitions for each posture) **CG:** maintain regular lifestyle	**EG:** 60 min/session; 3 sessions/week	12 weeks	Global cognitive function (MoCA); executive function (TMT-B)
10	Thomas et al. (38)	America	Age: 55–80 years	47/47%	**EG:** 15 **CG:** 15	**EG:** moderate to vigorous AE **CG:** stretching and toning	**EG&CG:** the former 10 weeks: 25–30 min/session; 3 sessions/week; 11–26 weeks: 30–35 min/session; 3–4 sessions/week; the latter 22 weeks: 30–40 min/session; 4–5 sessions/week	12 months	Delayed recall (WMS-R)

TC, Tai Chi; EG, experimental group; CG, control group; WMS-R, Wechsler memory scale-revised; AE, aerobic exercise; RE, resistance exercise; MMSE, mini-mental state examination; ADAS-cog, Alzheimer's disease assessment scale-cognitive subscale; DST, digit span test; DST-B, digit span test-backward; DST-F, digit span test-forward; RM, repetition maximum; MoCA, montreal cognitive assessment; RBMT, rivermead behavioral memory test; SDST, symbol-digit substitution test; SCWT, stroop color and word test; TMT-B, trail making test parts B; TMT-B–A, trail making test B minus A; PA, physical activity; WMS-LM I, logical memory I subtest of the Wechsler memory scale-revised; WMS-LM II, logical memory II subtest of the Wechsler memory scale-revised; TEA, test of everyday attention.

Among these 10 studies, three studies (30%) conducted multi-component interventions (e.g., exercise interventions combined with psychological cognitive interventions). The other seven studies (70%) conducted single-component interventions (i.e., the isolated exercise intervention). For multi-component interventions, two studies performed the aerobic exercise (AE), resistance exercise (RE), postural balance retraining, and dual-task training programs. Another study performed physical activity (PA) promotion and behavior modification, AE, and dual-task training programs. For single-component interventions, subjects in two studies received the AE training. Subjects in two studies performed the RE training and subjects in three studies received other forms of the exercise (e.g., Tai Chi and traditional Thai exercise).

**Figure 2:**
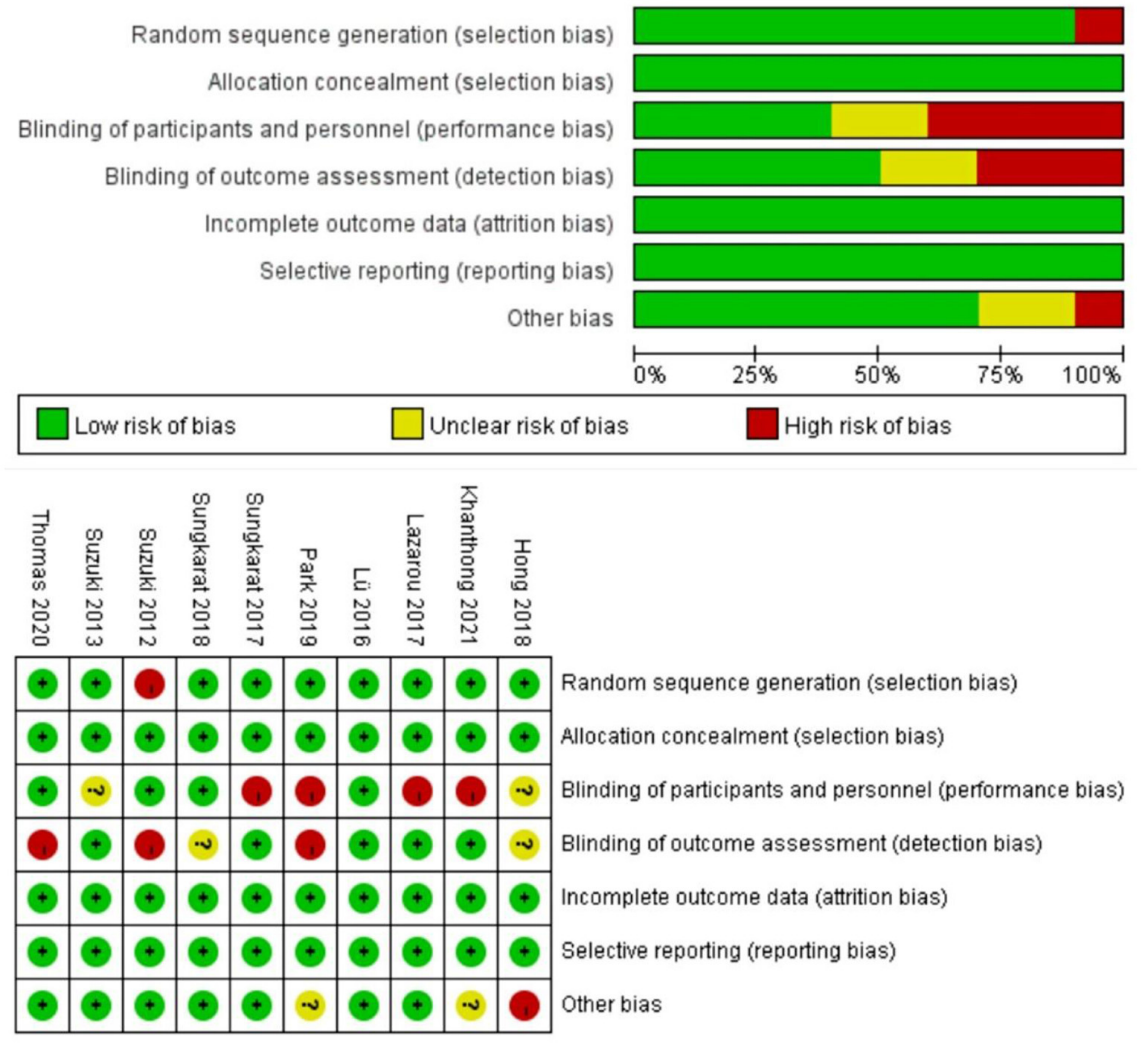
Details of bias of the included studies.

Additionally, the quality of included studies was evaluated according to the guidelines established by Higgins (34). As shown in Figure 2, these studies have relatively high qualities and the greatest source of bias was the blinding of participants and personnel (performance bias).

### Global cognitive function

**Figure 3:**
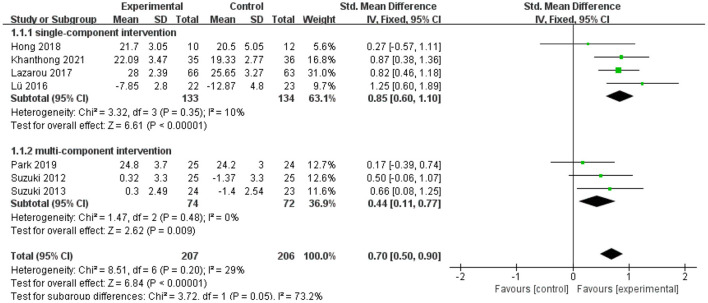
The influence of exercise interventions on global cognitive function.

Seven data points from seven studies reported the influence of exercise interventions on global cognitive function in aMCI individuals. As depicted in Figure 3, there was a significant difference between the experimental and control groups based on a fixed-effect model (SMD = 0.70; 95% CI = 0.50–0.90; *p* < 0.00001; *I*^2^ = 29%). Except that, a subgroup analysis was performed based on the forms of interventions (i.e., the single-component intervention and the multi-component interventions). Figure 3 presents that the single-component intervention is effective for aMCI patients to enhance the global cognitive function (SMD = 0.85; 95% CI = 0.60–1.10; *p* < 0.00001; *I*^2^ = 10%). And the multi-component interventions are also conducive to improving the global cognitive function (SMD = 0.44; 95% CI = 0.11–0.77; *p* = 0.009; *I*^2^ = 0%). According to the effect size in different intervention forms, the single-component intervention (SMD = 0.85) seems have a better improvement effect on global cognitive function than multi-component interventions (SMD = 0.44).

### Working memory

**Figure 4:**
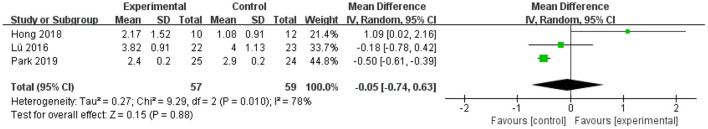
The influence of exercise interventions on working memory.

Three data points from three studies were synthesized to describe the influence of exercise interventions on working memory in aMCI individuals. As depicted in Figure 4, no significant difference was found between the experimental and control groups based on a random-effect model (WMD = −0.05; 95% CI = −0.74 to 0.63; *p* = 0.88; *I*^2^ = 78%).

### Immediate recall

**Figure 5:**
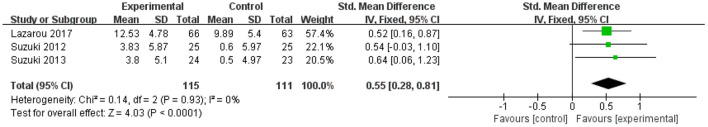
The influence of exercise interventions on immediate recall.

Three data points from three studies were combined to evaluate the influence of exercise interventions on immediate recall in patients with aMCI. As shown in Figure 5, there was a significant difference between the experimental and control groups based on a fixed-effect model (SMD = 0.55; 95% CI = 0.28–0.81; *p* < 0.0001; *I*^2^ = 0%).

### Delayed recall

**Figure 6:**
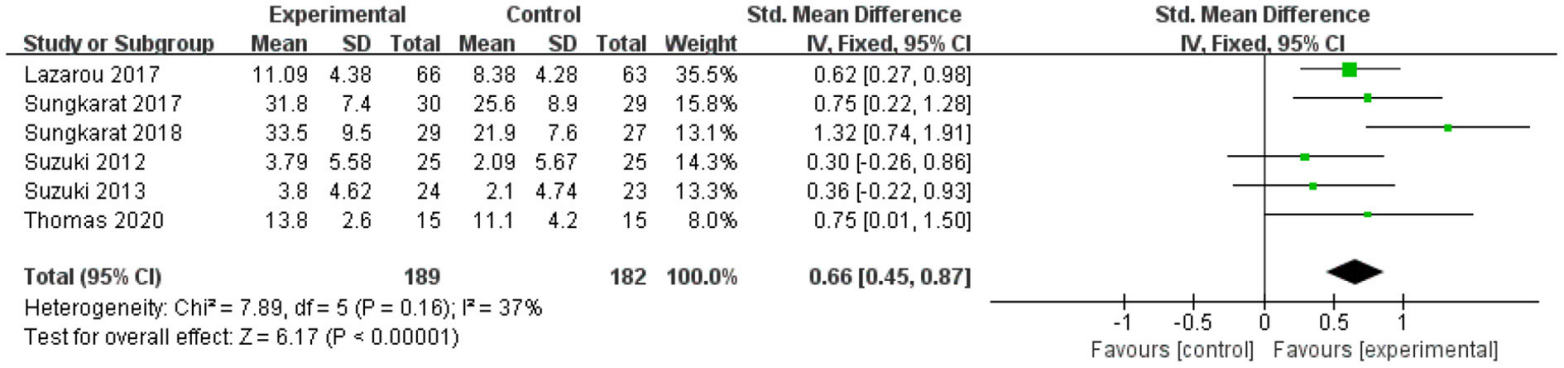
The influence of exercise interventions on delayed recall.

Six data points from six studies were pooled to investigate the influence of exercise interventions on delayed recall of patients with aMCI. As shown in Figure 6, there was a significant difference between the experimental and control groups based on a fixed-effect model (SMD = 0.66; 95% CI = 0.45–0.87; *p* < 0.00001; *I*^2^ = 37%).

### Executive function

**Figure 7:**
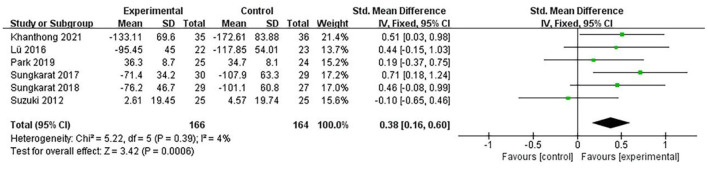
The influence of exercise interventions on executive function.

Six data points from six studies were merged to evaluate the influence of exercise interventions on the executive function of individuals with aMCI. As depicted in Figure 7, there was a significant difference between the experimental and control groups based on a fixed-effect model (SMD = 0.38; 95% CI = 0.16–0.60; *p*= 0.0006; *I*^2^ = 4%).

### Attention

**Figure 8:**
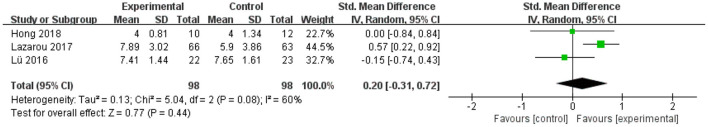
The influence of exercise interventions on attention.

Three data points from three studies were integrated to assess the influence of exercise interventions on attention in patients with aMCI. As shown in Figure 8, no significant difference was found between the experimental and control groups based on a random-effect model (SMD = 0.20; 95% CI = −0.31 to 0.72; *p*= 0.44; *I*^2^ = 60%).

## Discussion

To the best of our knowledge, this is the first meta-analysis investigating the influence of exercise interventions on the cognitive functions of patients with aMCI. The findings of this review suggested that exercise interventions had a positive effect on enhancing global cognitive function, immediate recall, delayed recall, and executive function of aMCI patients. And the single-component intervention seemed to show greater improvements in global cognitive function than multi-component interventions. By contrast, aMCI patients' several specific domains including working memory and attention could not be significantly improved by exercise interventions.

The findings demonstrated that exercise interventions could improve global cognitive function in patients with aMCI. The improvement of the global cognitive function induced by exercise interventions may be associated with neuroplasticity. Neuroplasticity is also known as brain plasticity, which refers to the ability of the brain to change, develop, and adapt functionally and structurally in response to environmental factors (47–49). Several studies have reported that exercise interventions can promote the process of neurogenesis, which directly leads to an increase in the number of new neurons (50–53). The increase of new neurons has been proved to be highly positive related to the enhancement of cognitive performance (54). Besides, exercise interventions are also helpful to enhance synaptic plasticity, which strengthens the connection between different nerve cells and may contribute to the improvement of cognitive levels (55, 56). Therefore, patients with aMCI may obtain significant benefits from exercise interventions in terms of global cognitive function.

Similar to exercise interventions, cognitive interventions are also proved to be conducive to increasing the new neurons' survival and guiding these new neurons to integrate into the existing brain networks (50, 51). Hence, we conducted a subgroup analysis based on the intervention forms (i.e., single-component interventions and multi-component interventions) and speculated that the multi-component interventions might bring a more obvious and comprehensive improvement in cognitions for aMCI individuals than the single-component intervention. However, the results were opposite to our initial hypothesis. Our results suggested that the single-component intervention might provide greater enhancement in global cognitive function than multi-component interventions. The poorer effect of the multi-component interventions may be related to the over-stress under dual-task stimulation. For patients with cognitive disorders, especially those old adults with aMCI, performing concurrent cognitive tasks while doing exercises is a great challenge (e.g., subjects were required to invent their poems while walking). Facing the dual challenge of physical exercise and mental tasks may prompt them to experience the excessive pressure, which weakens the cognitive benefits (21). This might partly explain that multi-component interventions may be less effective for aMCI patients to improve global cognitive function than the single-component intervention.

In addition, our results showed that exercise interventions were useful to improve some specific domains of cognitive functions including immediate recall and delayed recall of patients with aMCI. The enhancement of memory might be affected by the activation of noradrenergic. One study has found that both normal old individuals' and aMCI patients' memory can be consolidated due to the significant elevation of endogenous norepinephrine induced by a 6-min aerobic stationary bicycle exercise at 70% VO_2_max (57). Alternatively, it is well-known that the hippocampus is also involved in memory consolidation (58, 59). Some researchers have suggested that even mild physical exercise can facilitate the functional connection between the dentate gyrus/CA3 of the hippocampus and cortical regions (e.g., parahippocampal), which may contribute to memory improvement (60). Another explanation may be associated with the size of the hippocampus increasing after exercise interventions. It has been implied that exercise interventions (e.g., moderate-intensity AE) are able to enlarge the hippocampal volume and lessen memory decline in late adulthood (61).

Another finding in this meta-analysis was exercise interventions could improve the executive function of patients with aMCI. The better performance of executive function has been proved relevant to prefrontal cortex activation (62–64). An RCT study manifested that an AE intervention could increase bilateral prefrontal cortex activity, which contributed to a great improvement in executive function (63). Similarly, Chen et al. (62) adopted the near-infrared spectroscopy (NIRS) technique and indicated that a Chinese traditional exercise intervention Baduanjin lasting 8 weeks (90 min/day, 5 days/week) could promote a significant increase in oxygenated hemoglobin in the left prefrontal cortex and enhance executive function. Furthermore, moderate-vigorous-intensity PA has been demonstrated to be effective to ameliorate executive function by increasing the alpha band power and reducing beta-3 band power significantly (65). Hence, it may be possible to interpret the reason why the exercise is helpful to increase the performance of the executive function.

Exercise interventions couldn't alleviate the decline of working memory and attention in patients with aMCI. In the included articles that reported the outcomes of working memory and attention, the most common intervention duration was 12 weeks. Compared to other longer intervention periods related to other cognitive function domains (e.g., Park et al. conducted a 24-week intervention for improving executive function, and Lazarou et al. performed a 40-week intervention for preventing immediate recall and delayed recall decline), exercise interventions lasting 12 weeks may not be sufficient to improve working memory and attention. Therefore, exercise interventions beyond 12 weeks might be better. But further scientific evidence is needed to find a protocol (minimal or optimal) that is effective in enhancing cognition when providing exercise interventions in aMCI. In addition, it is believed currently that the enhancement of working memory mainly depends on visual and auditory training rather than exercise interventions. A previous meta-analysis (33) assessing the effect of the RE on working memory in mixed samples including aMCI individuals and naMCI individuals also obtained similar results that RE had no effect on improving working memory. The isolated AE didn't play a significant role in enhancing the working memory of people without dementia as well (66). Thus, it is possible that the benefits of exercise interventions for different cognitive domains are selective. Depending on different cognitive domains, exercise interventions should be selectively applied.

To the best of our knowledge, this is the first systematic review and meta-analysis evaluating the influence of exercise interventions on cognitive functions in one of the specific subtypes of MCI (i.e., patients with aMCI). Nevertheless, there are also some inevitable limitations in our study. First, we didn't use the funnel plot analysis to evaluate the publication bias due to the small number of studies for each cognitive outcome (fewer than 10 studies). Second, although aMCI consisted of the single-domain amnestic subtype and the multiple-domain amnestic subtype, further subgroup analysis was not performed, because few included articles in our meta-analysis distinguished the subtypes of aMCI. Third, we did not explore the effect of exercise interventions on other cognitive domains beyond the reported results, which was attributed to the limited included articles. Fourth, some cognitive domains have only been investigated by very few studies (e.g., working memory, immediate recall, and attention). So, any conclusion drawn (positive or negative) should still be speculative and more studies are likely needed to confirm the results. Finally, some unpublished articles or relevant studies using other languages except for English or Chinese may not be identified. This might reduce the comprehensiveness of this study.

## Conclusion

This review and meta-analysis demonstrates that exercise interventions are helpful for patients with aMCI to improve global cognitive function and several specific cognitive domains, such as immediate recall, delayed recall, and executive function. And the single-component intervention seemed to show a greater effect on improving global cognitive function than multi-component interventions. On the contrary, aMCI patients' working memory and attention can't be enhanced by exercise interventions.

## Data availability statement

The original contributions presented in the study are included in the article/supplementary material, further inquiries can be directed to the corresponding author.

## Author contributions

XH, HL, and HR: conceptualization. RW and HZ: methodology and investigation. RW: formal analysis and writing-original draft preparation. TS, LX, and YL: resources. XH and RW: writing-review and editing and visualization. XH: supervision. All authors contributed to the article and approved the submitted version.

## Funding

This study was supported by the Fundamental Research Funds for the Central Universities (Grant Number: 2015YB002).

## Conflict of interest

The authors declare that the research was conducted in the absence of any commercial or financial relationships that could be construed as a potential conflict of interest.

## Publisher's note

All claims expressed in this article are solely those of the authors and do not necessarily represent those of their affiliated organizations, or those of the publisher, the editors and the reviewers. Any product that may be evaluated in this article, or claim that may be made by its manufacturer, is not guaranteed or endorsed by the publisher.
